# Macro-micro mechanisms of void formation and grouting strength reinforcement in concrete pavement slabs using FEM-DEM coupling method

**DOI:** 10.1038/s41598-025-20825-w

**Published:** 2025-10-22

**Authors:** Xiaoyong Zhang, Jianxu Long, Zhuan Wang, Yi Wang, Dong Ran

**Affiliations:** Department of Architectural Engineering, Guizhou Communications Polytechnic University, Guiyang, 551400 China

**Keywords:** Concrete pavement, Mesoscopic parameters, FEM-DEM coupling method, Force chain, Cracks, Engineering, Civil engineering, Structural materials

## Abstract

Growing traffic and heavier vehicle loads cause voids beneath concrete pavement, significantly degrading road performance and safety. This study aimed to investigate the mechanical behavior of concrete pavement with voids and evaluate the effectiveness of grouting reinforcement. Researchers calibrated concrete surface and base layer parameters using uniaxial compression simulations and then developed a numerical model of concrete pavement void formation utilizing the FEM-DEM coupling method. The structure experienced initial cracking at approximately 76.6 kN, the load continued to increase to a peak of 139 kN, and after the peak the curve entered a descending branch, indicating that the structure began to degrade after reaching its ultimate bearing capacity. Analysis of displacement, crack patterns, and force chains revealed shear bands developing on either side of the void. A simulated grouting reinforcement, using 9,800 particles (0.5 mm diameter), demonstrated significant improvement, with the peak strength reaching 220 kN, representing a 58.3% increase. Enhanced load transmission, crack and displacement distribution, and force chain transfer confirmed the effectiveness of grouting in strengthening structural performance. This research provides vital theoretical support for addressing concrete pavement void issues.

## Introduction

**Fig. 1:**
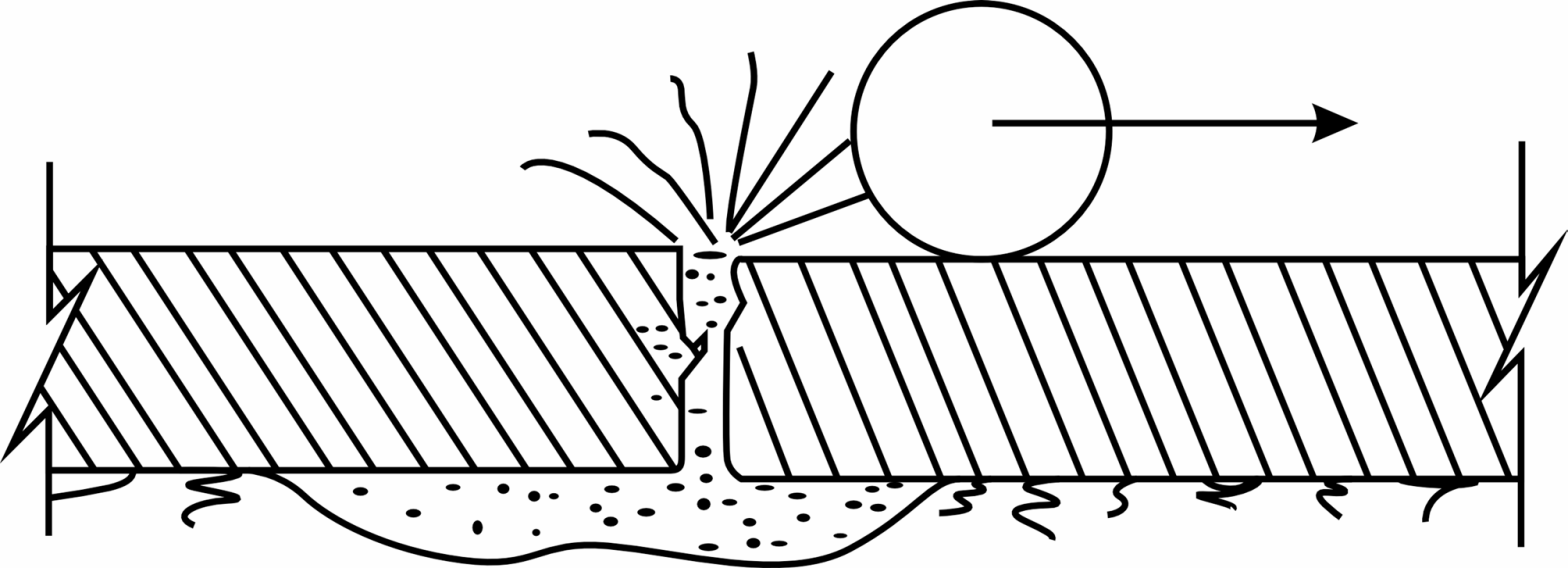
Mechanism of void formation under concrete pavement slabs.

Concrete pavements offer benefits like high load-bearing capacity, excellent stability, long service life, and low routine maintenance costs. They are one of the primary types of highway pavements, and an increasing number of countries are building cement concrete roads^1^. Recently, with the sharp rise in highway traffic and the increasing weight of vehicle axle loads, the existing concrete pavements, though still short of their design lifespan, have experienced varying degrees of damage and performance decline, significantly impacting road functionality and driving safety^1,2^. Common pavement defects in concrete include step-offs, fractures, fragmentation, and void formation. Almost all of these defects, such as step-offs, fractures, and fragmentation, are associated with void formation beneath the slabs^3–5^. In terms of the void formation mechanism, Hansen et al.^6^ suggested that the pump force at the bottom of the concrete slab is one of the primary causes of the void formation beneath the pavement. Cracking and destruction of highways are attributed to void formation beneath the slabs^7^. Void formation is thought to involve two main components: the first is the net settlement of fine aggregates at the bottom of the slab, and the second is the ejection of fine aggregates from beneath the pavement slab (Fig. 1). The concrete slab at the void location acts like a cantilever structure, experiencing unfavorable stress conditions. Under vehicle loads, it undergoes large stresses and deflection, making it highly susceptible to cracking. Over time, repeated vehicle loads cause it to fracture, resulting in a decline in the pavement slab’s performance and a reduced fatigue life^8^.

In real-world engineering applications, it is vital to understand the mechanism of void formation beneath the slab and the changes in macroscopic mechanical performance after void formation to formulate effective mitigation strategies^9,10^. Grouting refers to a technique where a solidifiable liquid substance is injected into rock fractures via a grouting pump or similar equipment, aiming to enhance the physical and mechanical attributes of fractured rock masses^11^. Engineering applications have demonstrated that this approach efficiently boosts the strength, structural integrity, and stability of fractured rock formations. In recent years, numerous researchers have conducted fruitful investigations into grouting materials, leading to the development of various types with distinct properties^12–14^. These materials can be categorized primarily into inorganic cementitious grouts and organic chemical grouts. Additionally, both domestically and internationally, there is a lack of research on the mechanical state following grouting treatment for void formation beneath pavement slabs. The absence of a mechanical foundation for this treatment method hinders its development and practical engineering applications.

In 1979, Cundall et al.^15^ introduced the discrete element method (DEM) suitable for geomechanics. As a form of discrete element, the Particle Flow Code (PFC) divides the computational time into sufficiently small intervals, each representing a computational step, applying Newton’s second law and force-displacement relations at each step^16^. The fundamental unit of PFC is the particle, which provides a distinct advantage in simulating damage behaviors such as cracking and fracture.

PFC defines three types of contact constitutive models: stiffness, sliding, and bonding. The bonding model can simulate the cementation between concrete particles, and the bonding fracture process can simulate the expansion of microcracks, revealing the propagation patterns of microcracks in concrete. There is a relatively larger body of research based on PFC regarding aggregate particle morphology reconstruction^17,18^, gradated mixture reconstruction^19,20^, and numerical simulation experiments, while literature using discrete element models to study concrete fragmentation is relatively scarce.

Although PFC is highly effective in simulating concrete, its use is limited by computational efficiency, and it is generally suited for simulating small-scale laboratory experiments^21–23^. Su et al.^24^ used PFC to propose and test a discrete element model for concrete. Using the principles of orthogonal experimental design and response surface methodology, they conducted uniaxial compression tests to establish a mapping relationship between macroscopic and microscopic parameters of concrete. Pieralisi et al.^25,26^ utilized the DEM model to simulate the compaction process and generate the microstructure, predicting the mechanical properties of permeable concrete. They proposed a constitutive equation based on the discrete element method to model the interaction between aggregates in freshly mixed permeable concrete during compaction, with the aggregates encased in cement paste. The elastic and viscous components of the normal force between particles were calibrated based on the work of Shyshko and Mechtcherine^27^, and the accuracy of the constitutive equation was ultimately verified through experimental testing. Ansari and Mahajan^28^ employed a parallel bonding model to substitute the cement’s bonding with the aggregates and validated this approach through experiments.

This study focuses on the issue of microcracking in concrete pavements. It is difficult to study full-scale road structures using PFC, as the boundary wall units in PFC are rigid, which does not accurately represent real-world conditions. The DEM serves as a powerful numerical approach for simulating crack propagation and wear in granular materials. However, the high computational cost of DEM restricts its application to large-scale domains.To tackle this challenge, we utilize DEM to model regions undergoing crack propagation and wear, while employing the Finite Element Method (FEM) for areas with minor deformations. Therefore, it can be seen that the continuous-discrete coupling method effectively combines the advantages of the discrete element method and finite element (or finite difference) method. This method is widely used in various fields and is a rational and efficient approach in numerical simulations^29^. The FEM-DEM coupling method is employed to simulate processes such as compression and fracture in concrete pavements by defining bonding and particle morphology, investigating the micro-damage characteristics of concrete pavements. A parallel bonding model is used to substitute concrete, and the concrete damage characteristics are explored by constructing a micro-scale DEM model^30,31^.

This study uses the FEM-DEM coupling method to conduct a comparative analysis of the bottom voiding and grouting reinforcement of concrete pavements. Initially, uniaxial compression simulations were employed to calibrate the mesoscopic parameters of both the surface and base layers of the concrete. Following this, a numerical model of the pavement structure was developed, and a comparison was made of the macroscopic mechanical properties and mesoscopic variations such as particle displacement, force chains, and crack development after voiding and grouting reinforcement. The findings offer a theoretical foundation for the study of treatment methods for bottom voiding and grouting in concrete pavement slabs.

## Discrete element modeling and mesoscopic parameter calibration of bottom voiding

### Calculation model

**Fig. 2:**
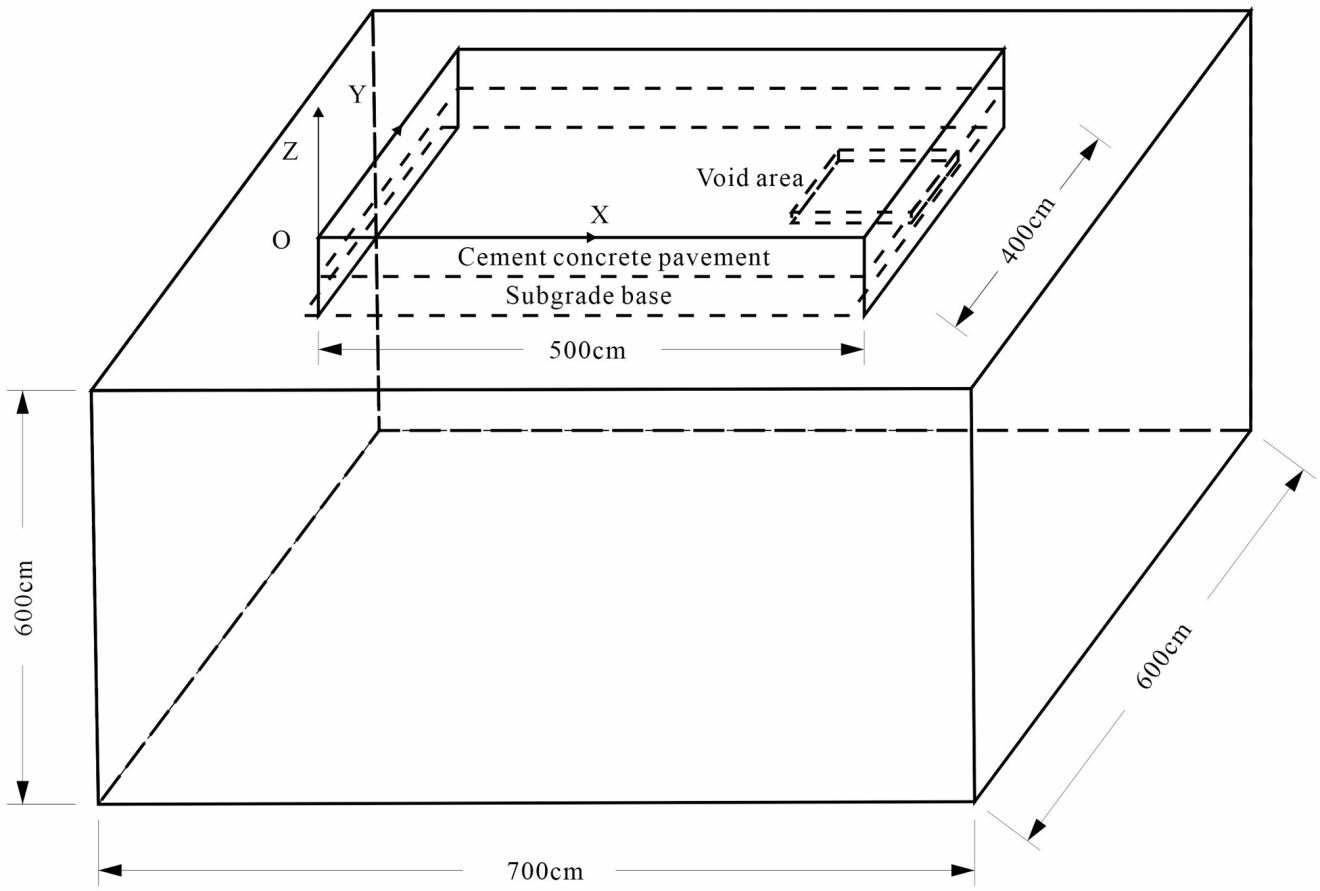
Calculation Model of Bottom Voiding in Concrete Pavement.

The calculation model for the bottom voiding of the concrete pavement is illustrated in Fig. 2. The dimensions of the computational model were referenced from the numerical model sizes presented in literature^21^. This selection was made to ensure computational efficiency while simultaneously avoiding model size effects during the simulation. This choice guarantees the accuracy and generalizability of the results. The concrete panel has a length of 5 m, a width of 4 m, and a thickness of 0.26 m. The base layer is 0.5 m thick, and the foundation is expanded to dimensions of 7 × 6 × 6 m. The voiding area is positioned at the bottom of the concrete panel, with dimensions of 1 × 1 × 0.01 m. The primary calculation parameters for each structural layer are provided in Table 1.

**Table 1:** Key calculation parameters for each structural Layer.

Structural Layers	Thickness (cm)	Young’s Modulus E (MPa)	Poisson’s Ratio
Concrete Slab	26	30,000	0.15
Grouted Block	1	1000	0.25
Subgrade Base	50	1200	0.20
Ground Subgrade	–	300	0.40

**Fig. 3:**
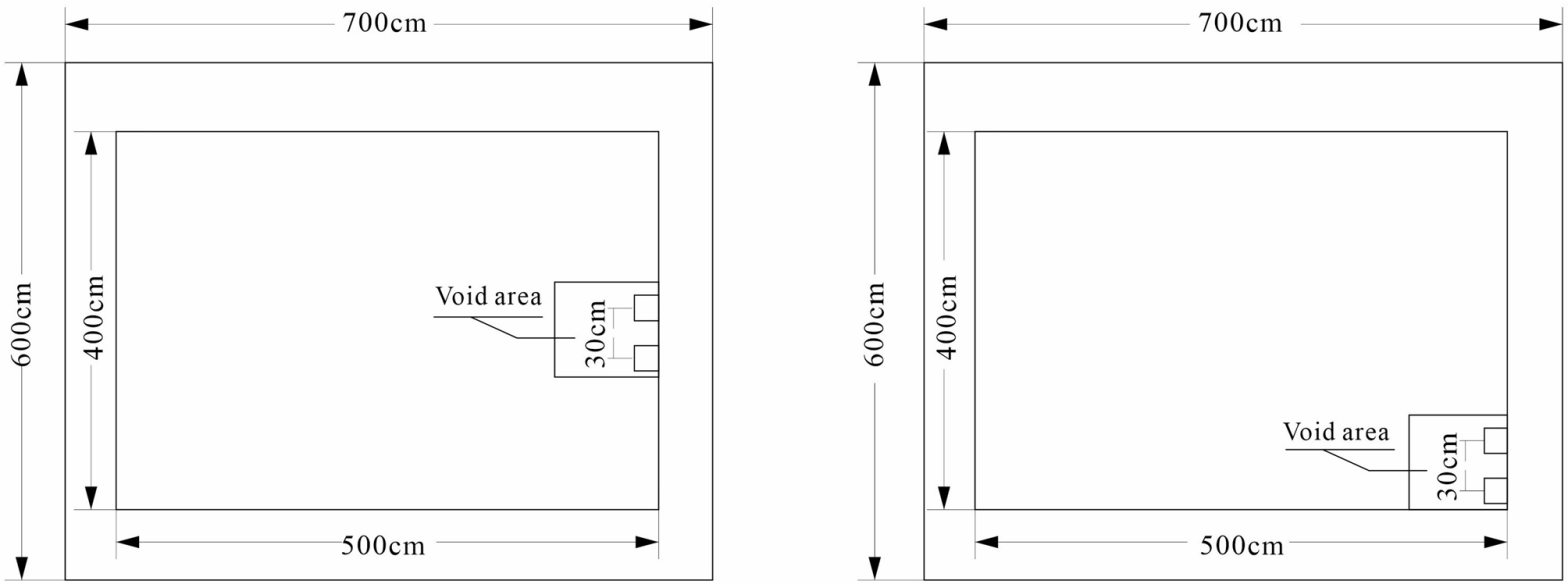
Load distribution for bottom voiding calculation.

The vehicle load is represented by a single wheel pressure with a 20 × 20 cm contact area, and the wheel spacing is 30 cm. Through calculations and comparison of different loading positions, it was observed that when the pavement slab edge is grouted, the wheel load symmetrically applied at the bottom plate on one side of the slab is most detrimental to the stress distribution of the pavement. The loading arrangement is shown in Fig. 3.

### Principle of continuous-discrete coupling approach

In coupled simulations, when the discrete element method (DEM) is used to model macroscopic engineering, the number of particles required is large and the calculations are time-consuming. The continuous-discrete coupling method can help alleviate this problem by reserving a void region in the continuous element core section, where DEM modeling is applied. Communication between continuous and discrete elements takes place through an interaction interface, thus ensuring both computational efficiency and accuracy^32,33^.

**Fig. 4:**
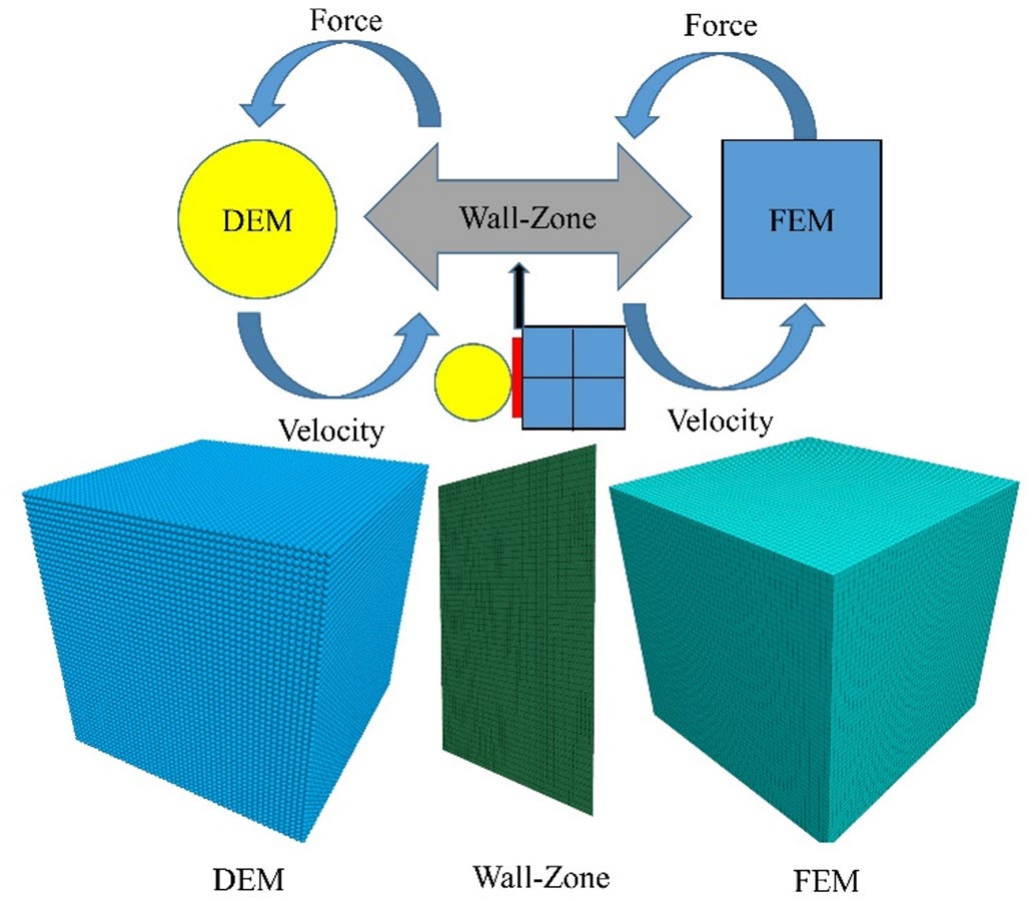
The principle of FEM-DEM coupling.

The principle of FEM-DEM coupling is illustrated in Fig. 4. This approach uses walls/shells as interaction interfaces. Velocity data from the continuous element nodes are passed to the wall/shell unit nodes, where they are converted into unbalanced forces, which are then distributed to the DEM particles via shape functions. Likewise, the unbalanced forces in the DEM are converted into velocity and sent back to the continuous element nodes through the wall/shell unit nodes, continuing the cycle until dynamic equilibrium is reached. Once coupling is activated, the calculation must run under large deformation conditions, and to ensure the balance of forces and moments at the coupling interface, full computation mode should be activated^34^.

**Fig. 5:**
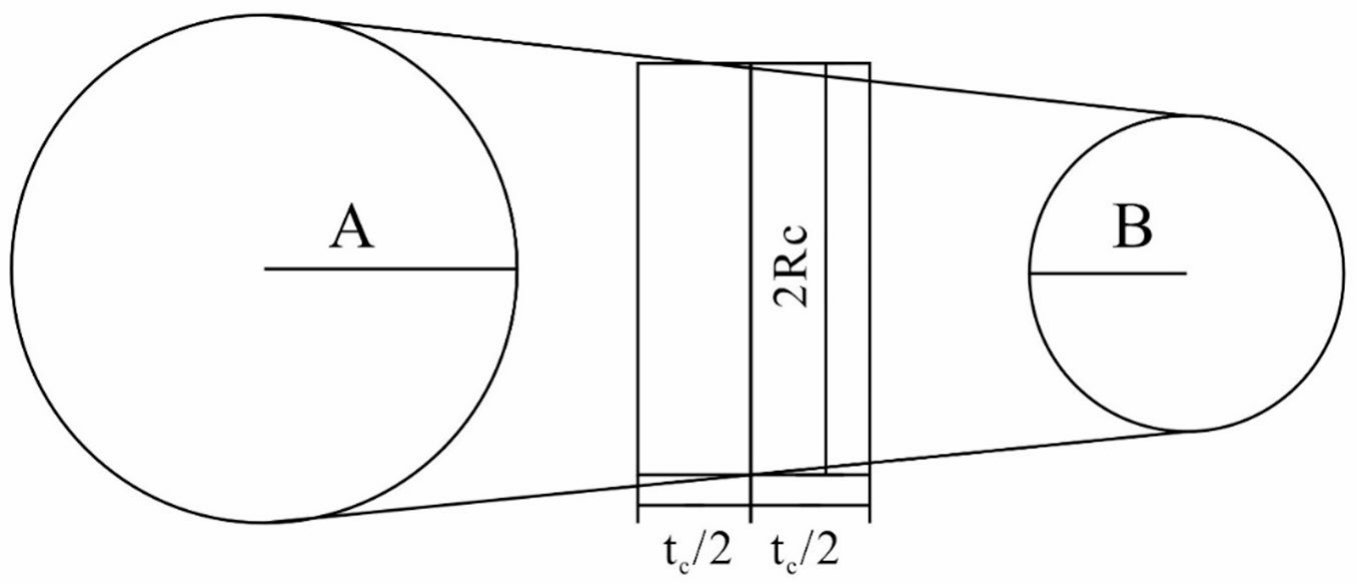
Schematic diagram of crack generation and propagation.

The contact bonding constitutive model in the particle flow method can effectively simulate the generation and development of cracks in the specimen. In the program, the cohesion between particles is determined by setting the tangential and normal bonding strengths and the friction coefficient. If the stress in any direction of the particle model exceeds its corresponding bonding strength during loading, the bonding between particles will fail, thus generating cracks. Cracks can only form during the loading process in the particle flow program if a cohesive contact model is applied between the particle bodies. Thus, the microscopic parameters set when assigning the contact model will significantly affect the number and position of the cracks that form in the model. The cohesion between particles can be considered as a cylindrical surface in the normal direction within the model plane, as shown in Fig. 5. The crack representation in this model is as follows^35^:

Assume that the two particles generating the crack are A and B, the thickness of the crack cylindrical surface can be expressed as:

1 $${t_c}=d - \left( {{R^{[A]}}+{R^{[B]}}} \right)$$

In the formula: *d* represents the distance between the two particles; ${R^{[A]}}$ represents the radius of particle A; ${R^{[B]}}$ represents the radius of particle B;

The central position of the cylindrical surface can be expressed as:

2 $${x_i}=x_{i}^{{[A]}}+\left( {{R^{[A]}}+\frac{{{t_c}}}{2}} \right){n_i}$$

In the formula: ${n_i}$ represents the normal direction $x_{i}^{{[A]}}$ from to ${R^{[A]}}$.

The radius of the cylindrical surface can be represented as:

3 $${R_c}={R^{[A]}}+({R^{[B]}} - {R^{[A]}})({R^{[A]}}+\frac{{{t_c}}}{2})/d$$

As described above, in the particle flow program, cracks in the model can be represented by parameters such as thickness, radius, normal direction, and center point location. The thickness can be represented by the gap between the two particles, the radius can be represented by the length of the mid-plane of the connecting cylindrical surface between the particles, the crack normal direction aligns with the direction of the line connecting the centers of the two particles, and the center point is the intersection of the centerline between the particles and the positions of the two particle centers^36^. Therefore, in numerical simulations, by recording the type of damage when the contact bond breaks, it is possible to overcome the limitations of laboratory and field in-situ tests, which are often unable to observe and differentiate between tensile damage and shear damage.

### Calibration of discrete element mesoscopic parameters

**Fig. 6:**
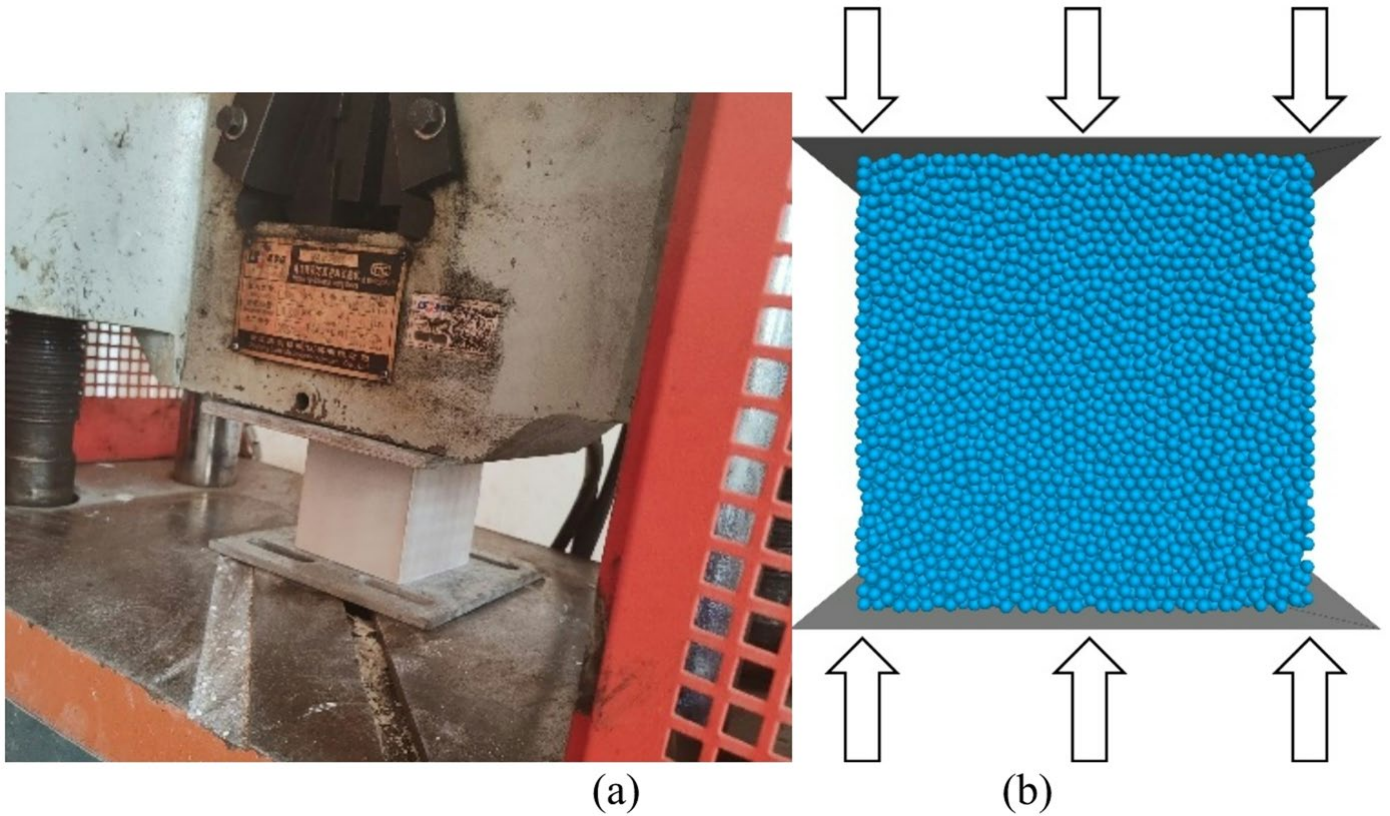
Compression tests and discrete element calculation model of standard concrete test blocks. (**a**) Laboratory tests, (**b**) Discrete Element Model.

**Fig. 7:**
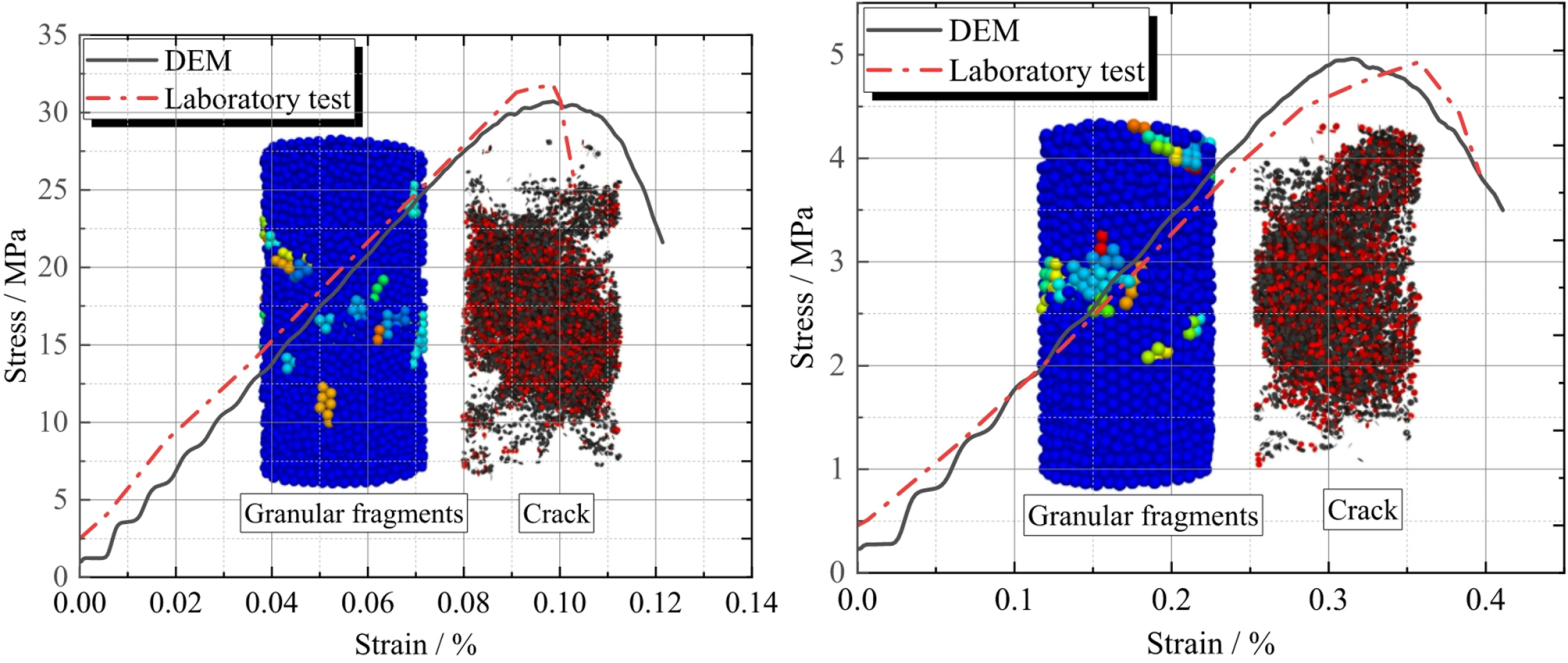
Stress-strain curves of uniaxial compression test. (**a**) Concrete surface layer (**b**) Base course.

In the discrete element method, the macroscopic mechanical properties of the material are governed by the micro-parameters of the particles and the contact constitutive model^37^. In this study, uniaxial compression tests are conducted to obtain the physical and mechanical property indicators of the discrete element model. The uniaxial compression test was conducted using a servo-hydraulic testing machine with a maximum loading capacity of 3000 kN. The machine is equipped with a high-precision load cell and a linear variable differential transformer (LVDT) for measuring axial deformation, with a resolution of 0.001 mm. Standard concrete test blocks were selected to conduct uniaxial compressive mechanical tests. The standard cubic test blocks with the size of 100 × 100 × 100 mm were used as the key specimens for the uniaxial compressive tests. During the test, the axial load and corresponding axial deformation were continuously recorded until the specimen failed. For each of the three groups, the compressive strength, elastic modulus, and peak strain were calculated based on the recorded data. To ensure the reliability and reproducibility of the results, the final test results (including compressive strength, elastic modulus, and peak strain) were determined as the average value of the three groups of test results. This averaging method helps to eliminate random errors caused by minor variations in specimen preparation and testing processes, providing more representative and robust data for subsequent analysis. The test model of the standard concrete test block and the discrete element calculation model are shown in Fig. 6, which provides a simulation basis for the subsequent in-depth numerical analysis and study of mechanical properties. The particle size and micro-parameters of the uniaxial specimen are consistent with the foundation parameters. Rigid wall boundaries are used as the upper and lower loading plates for the specimen, with a loading rate of 0.01 mm/s, and the normal and tangential contact stiffness are identical to the specimen’s stiffness. After multiple trial calculations, the mesoscopic parameters are as shown in Table 2, and the stress-strain curves of different models are as shown in Fig. 7.

**Table 2:** Mesoscale parameters for various geological Layers.

Structural Layers	Cement Concrete Pacement	Subgrade base
Particle contact model	linearpbond	
Effective modulus (Pa)	50 × 10^9^	1 × 10^9^
Normal-to-shear stiffness ratio	1	1
Bond effective modulus (Pa)	50 × 10^9^	1 × 10^9^
Bond normal-to-shear stiffness ratio	1	1
Tensile strength (N)	15 × 10^6^	11.2 × 10^5^
Cohesion (N)	15 × 10^6^	11.2 × 10^5^
Friction angle (°)	0	10
Friction coefficient	0.5	0.3
Particle density (kg/m^3^)	2800	2500

Comparison and validation between DEM and laboratory tests in the research on the concrete surface layer, uniaxial compression tests were conducted both through DEM simulations and laboratory experiments, yielding the corresponding stress-strain curves as shown in the Fig. 7a. A comparative analysis of the curve profiles reveals a high degree of consistency between the DEM simulation curve and the laboratory test curve. During the stress development phase, both curves exhibit a similar pattern: stress increases progressively with strain, reaches a peak value, and then declines. Focusing specifically on the peak strength, both the laboratory tests and DEM simulations yield a value of approximately 30 MPa, demonstrating excellent numerical agreement. This consistency strongly validates the rationality and effectiveness of the mesoscopic parameters employed in the DEM simulations. In DEM modeling, mesoscopic parameters are fundamental for constructing the model and accurately representing the material’s mechanical behavior. The close match between the simulation results and the actual laboratory test data, particularly in such a critical mechanical peak strength and the overall curve trend, indicates that these parameters can precisely capture the mechanical response characteristics of the concrete surface layer under uniaxial compression. Consequently, they provide a reliable data foundation and parameter basis for subsequent DEM-based simulations under more complex conditions, enabling in-depth investigations into the evolution of the concrete surface layer’s mechanical properties under various conditions.

For the base layer, stress - strain curves from DEM simulation and lab uniaxial compression test are shown in the Fig. 7b. As strain rises from 0, both curves DEM: solid line; dashed line show stress increasing, reflecting internal force mobilization under compression. Both DEM and lab tests give a peak strength of 5 MPa, with peak strain at 0.32%. This high consistency validates the rationality of mesoscopic parameters in DEM.

### Establishment of the FEM-DEM coupling model

**Fig. 8:**
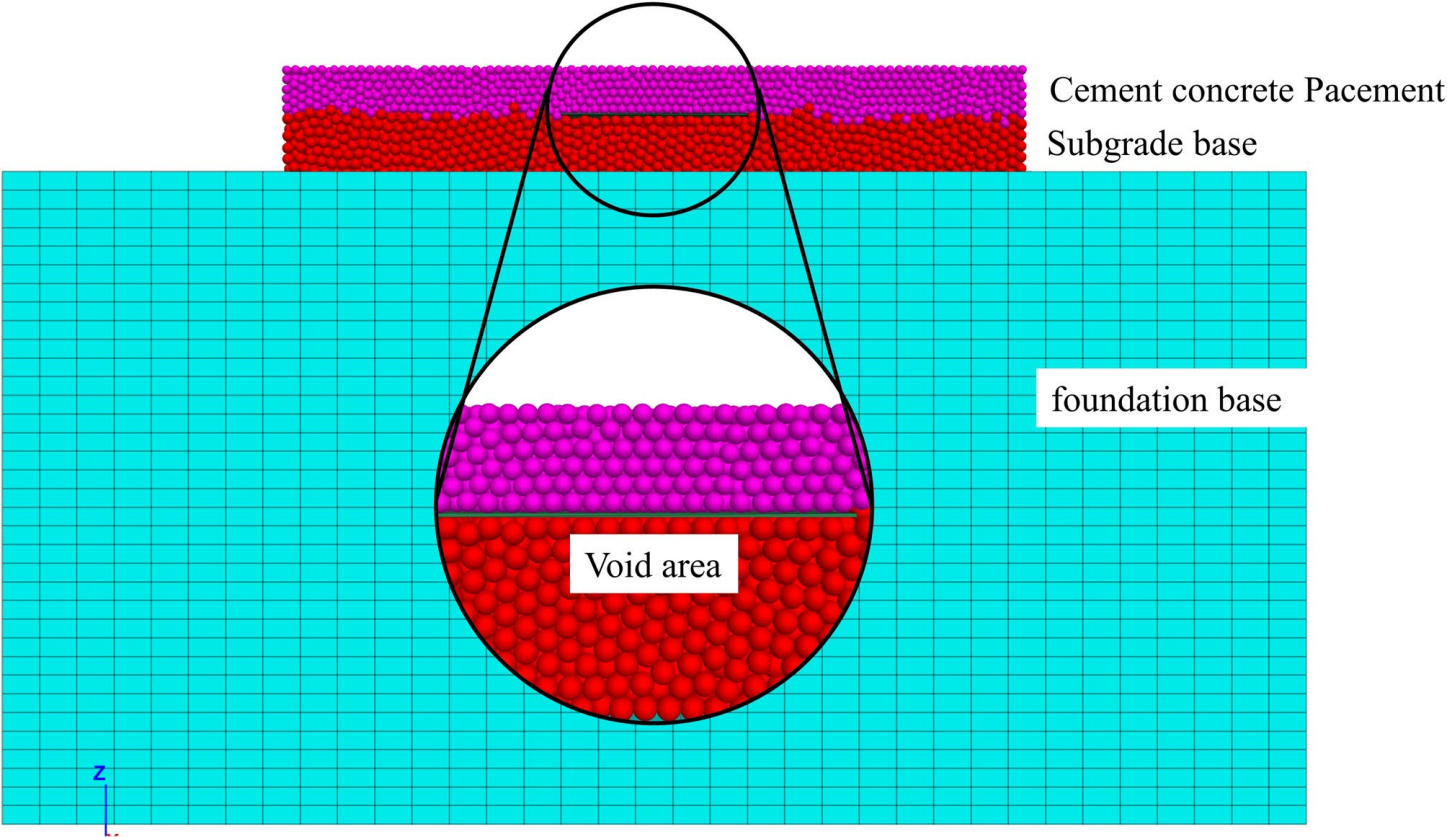
FEM-DEM Coupling Model for Concrete Pavement Base Slab Void.

The FEM-DEM coupled model for the bottom void of the concrete pavement is shown in Fig. 8. The subgrade consists of particles with a radius of 3 cm, and the concrete slab consists of particles with a radius of 2.5 cm. The model is composed of a large number of particles, totaling 125,900, with 59,587 particles in the concrete surface layer and 66,314 particles in the subgrade. This model is developed using the DEM and requires validation before use to ensure its reliability for further research. Choosing the appropriate particle radius ensures the accuracy of the simulation results. A larger particle radius reduces computation time but may ignore some microscopic mechanical behaviors, while a smaller radius provides more accurate simulation of micro-mechanical behaviors at the cost of increased computational cost. The PFC is used to simulate the behavior of granular materials, reflecting the mechanical properties of the material through particle interactions. The subgrade is modeled with a finite element mesh, which helps improve computational efficiency^38^. The parameters are shown in Table 3. The FEM-DEM coupling method combines the benefits of the FLAC and the PFC. The FEM is advantageous for continuous media and large deformation problems, while the DEM is excellent for simulating granular materials and crack propagation. This coupled method enables a more comprehensive simulation of complex geotechnical engineering problems^39^.

**Table 3:** FEM parameters of the subgrade.

Structural Layers	Model	Density (kg/m^3^)	Young’s Modulus (MPa)	Poisson’s Ratio	Bulk Modulus (MPa)	Shear Modulus (MPa)
Subgrade	Elastic Model	2300	300	0.4	500	107.4

For the discrete element model, the boundaries are defined as free boundaries. This means that when the model is subjected to external loads, the particles within the model are allowed to move freely in the spatial domain without any constraints, which accurately simulates the actual mechanical behavior of the material under unconstrained conditions. In terms of the mesh boundaries, specific constraints are applied to ensure the stability of the computational domain during the simulation. The surrounding boundaries of the mesh are constrained in the x and y directions, preventing any displacement in these two directions, while the z direction remains unconstrained. The bottom boundary of the mesh is fully constrained in all x, y, and z directions to fix it in space, providing a stable foundation for the entire simulation system.

When constructing the coupling model, the core research area is set as a discrete domain, and DEM is used to depict mesoscopic cracking and block movement; the area with gentle deformation and uniform mechanical response is set as a continuous domain, and FEM is used to efficiently calculate stress and strain, achieving “refinement of the core and high efficiency of the periphery”. To ensure the reliability of the finite element (FEM) results for the foundation layer (a key part of the FEM-DEM coupling model) and balance computational accuracy and efficiency, a mesh sensitivity analysis was conducted as a critical preliminary step before formal simulation.

**Fig. 9:**
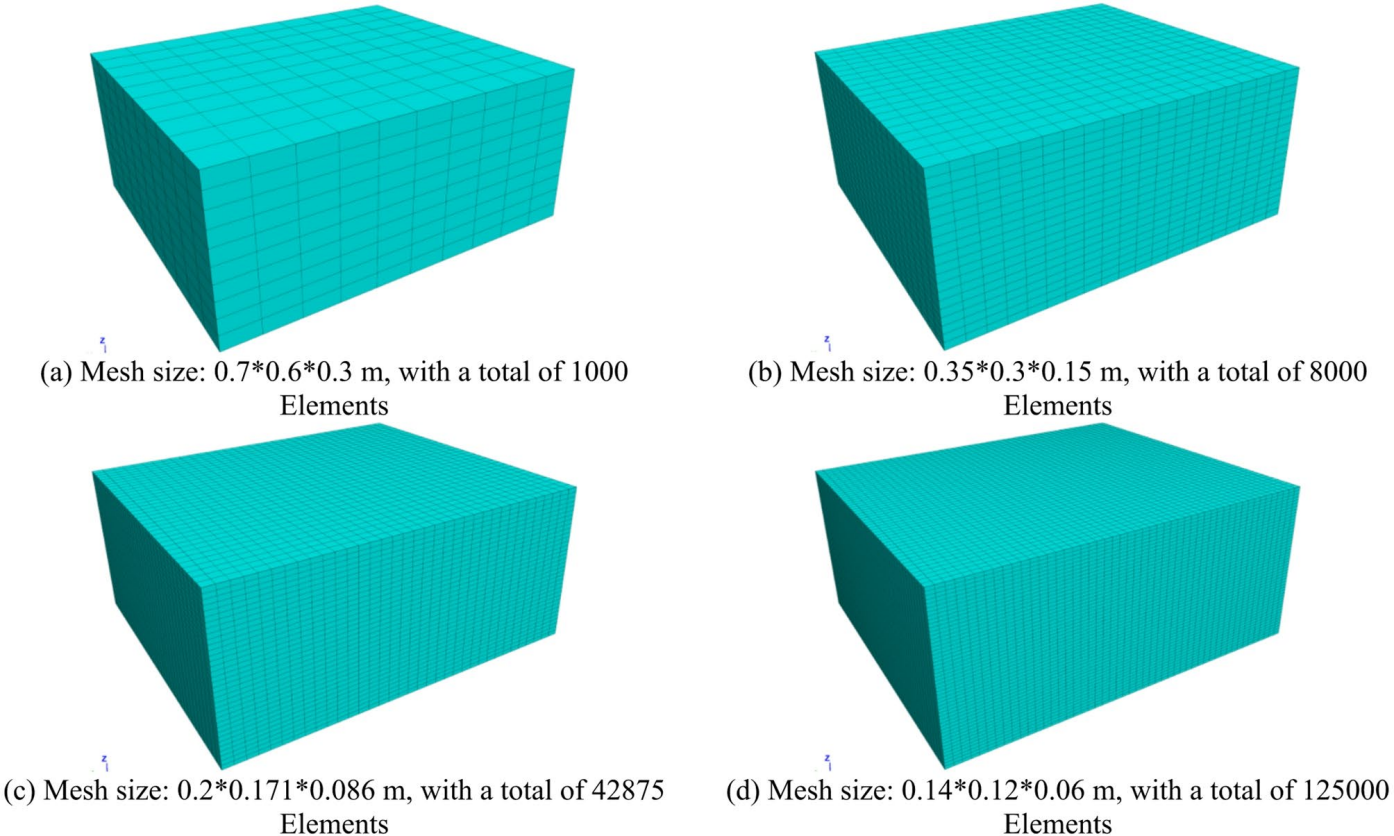
FEM-DEM coupling model for concrete pavement base slab void.

The mesh sensitivity analysis in this study focuses on the foundation layer (FEM domain), with the settlement of the most central mesh after the upper particles exert pressure on the mesh under self-weight as the reference index. We have designed four groups of schemes with different mesh sizes to compare the computational accuracy (difference between relative error and theoretical value) and computational efficiency (computational time). The specific parameters of each scheme are detailed in Fig. 9; Table 4.

**Table 4:** Mesh sensitivity analysis scheme and results.

Group	Mesh Size of Foundation Layer (m)	Number of Finite Elements	Simulated Vertical Displacement (mm)	Relative Error (%)	Computational Time (h)
1	0.7*0.6*0.3	1000	0.138	9.21	2.1
2	0.35*0.3*0.15	8000	0.145	4.61	3.8
3	0.2*0.171*0.086	42,875	0.151	0.66	6.3
4	0.14*0.12*0.06	125,000	0.152	0	14.7

Based on the above comprehensive evaluation, with the results of Group 4 as the reference standard, Group 3 demonstrates a relatively small relative error (0.66%) while significantly reducing computational resource consumption (with 66.7% fewer finite elements and 57.1% less computational time compared to Group 4). This balance between accuracy and efficiency is particularly critical for large-scale FEM-DEM coupling simulations, as it avoids excessive computational burdens caused by over-pursuit of precision while ensuring that the simulation results meet engineering analysis requirements. Therefore, considering both the reliability of research data and the practicality of computational implementation, this study ultimately selects the mesh size of Group 3 as the optimal unit size for the foundation layer.

The establishment of the void model employs an efficient approach. Initially, a wall is created in the void region. The role of this wall is to maintain the model’s stability at the initial stages of model setup. Once the model reaches equilibrium and appropriate microscopic parameters are assigned, the wall is deleted to create the void region. The benefit of this approach is that after the model achieves equilibrium, the wall is removed to create the void region. At this stage, the model is stable, allowing for further calculations to be carried out based on this stability, thus reducing the number of iterations and computational load.

This paper uses a rigid wall as the loading plate for the subgrade model. Based on the research results of Zhang et al.^40^, after several trial calculations, a loading rate of 0.005 mm/s was determined. This speed guarantees that the model undergoes quasi-static loading during the loading process, thus optimizing computational efficiency.

**Fig. 10:**
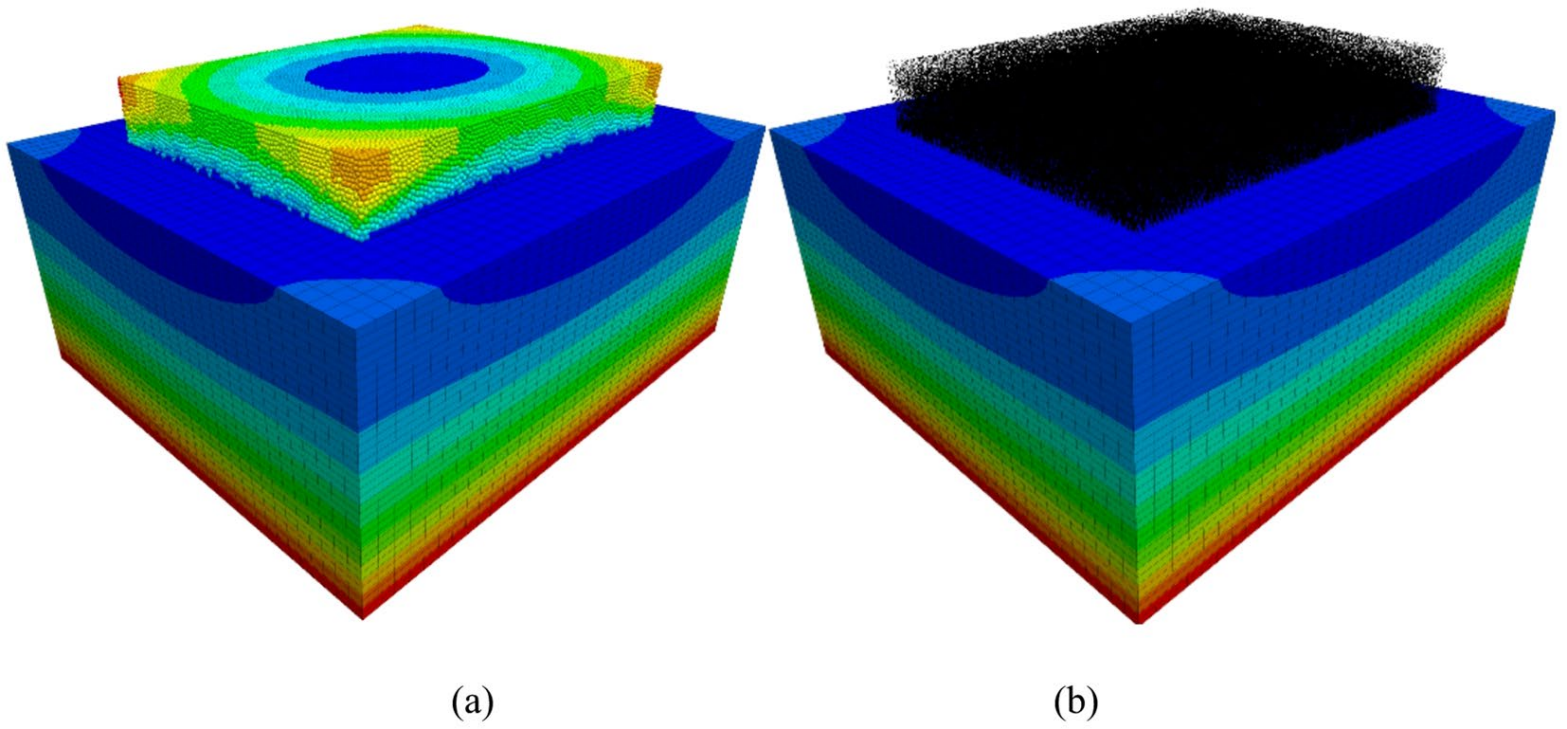
Gravity equilibrium of the discrete element model of the subgrade. (**a**) Displacement contour map, (**b**) Contact force chain distribution map.

Figure 10 shows displacement contour plot and force chain distribution of the DEM model after self-weight equilibrium. From the figure, it can be observed that the settlement displacement shows a roughly horizontal distribution. This implies that in this soil model, the settlement amounts at various horizontal layers are fairly uniform. This phenomenon is consistent with the property of uniform vertical compression of the soil under gravity. In actual engineering, this kind of horizontally distributed settlement displacement typically occurs in homogeneous soil layers. Under the influence of gravity, the soil layers compress uniformly downward, resulting in similar settlement amounts across each layer. The force chain shows a dense distribution in the lower section and a more sparse distribution in the upper section. This distribution pattern aligns with the actual soil’s initial stress distribution under the influence of gravity. Due to gravity, the lower layers of soil must bear the weight of the upper soil. As a result, the force chain between particles in the lower soil is more compact. The upper soil, however, experiences less pressure, resulting in a relatively sparse force chain.

The force chain shows a dense distribution in the lower section and a sparse distribution in the upper section. This distribution pattern aligns with the initial stress distribution in the soil under the influence of gravity. In the soil, due to gravity, the lower layers must support the weight of the upper layers. As a result, the force chain between particles in the lower soil is more compact. In contrast, the upper soil experiences less pressure, and the force chain is relatively sparse.

## Result

### Research on the loading mechanism of floor voiding

#### Variation laws of load and microcracks

**Fig. 11:**
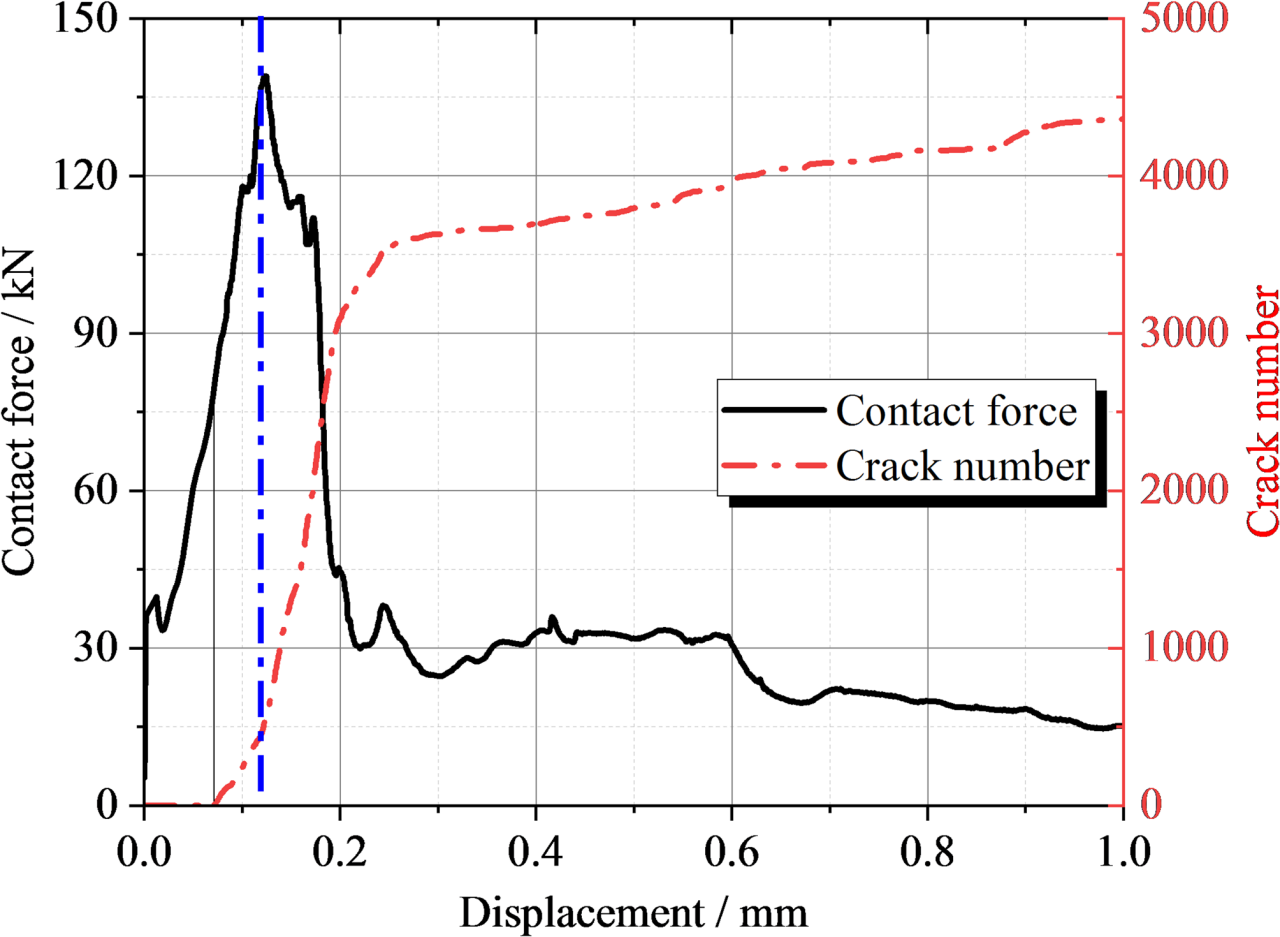
Curves of the void load of the road surface bottom plate and microcracks varying with displacement.

The variation in load and microcrack count with displacement during loading can be clearly observed from the Fig. 11. During the initial loading phase, the contact force increases rapidly with displacement until it reaches the peak load of 139 kN. At the same time, the number of cracks gradually increases, but prior to reaching the peak load, the increase in cracks is relatively slow, amounting to only 471 cracks. After the load surpasses the peak, the number of cracks begins to rise sharply. This suggests that once the ultimate bearing capacity is reached in concrete structures, internal microcracks rapidly propagate and connect, causing a gradual loss of structural integrity. When the strain reaches 0.26, the crack count tends to stabilize at approximately 3580. This indicates that at this strain level, the internal microcracks in the concrete have fully developed, creating a relatively stable crack network. The critical load at which microcracks first appear in the concrete structure is around 76.6 kN. Before the cracking load, the concrete structure remains largely in the elastic deformation phase. Because of the void, the load is concentrated at the edges of the void, rather than being evenly distributed across the entire pavement structure. This phenomenon of load concentration results in stress concentration at the edges of the void, triggering the formation and propagation of microcracks. At the peak load, stress at the edges of the void reaches its maximum, and microcracks expand rapidly. As displacement continues to increase, the crack network gradually becomes connected, causing a reduction in the structural load-bearing capacity. Once the strain reaches 0.26, the crack count stabilizes, due to the crack growth being restricted by the surrounding uncracked concrete. At this stage, the load is primarily carried by the uncracked concrete section and the formed crack network, with the structure entering a relatively stable plastic deformation phase.

#### Variation laws of the particle displacement field

**Fig. 12:**
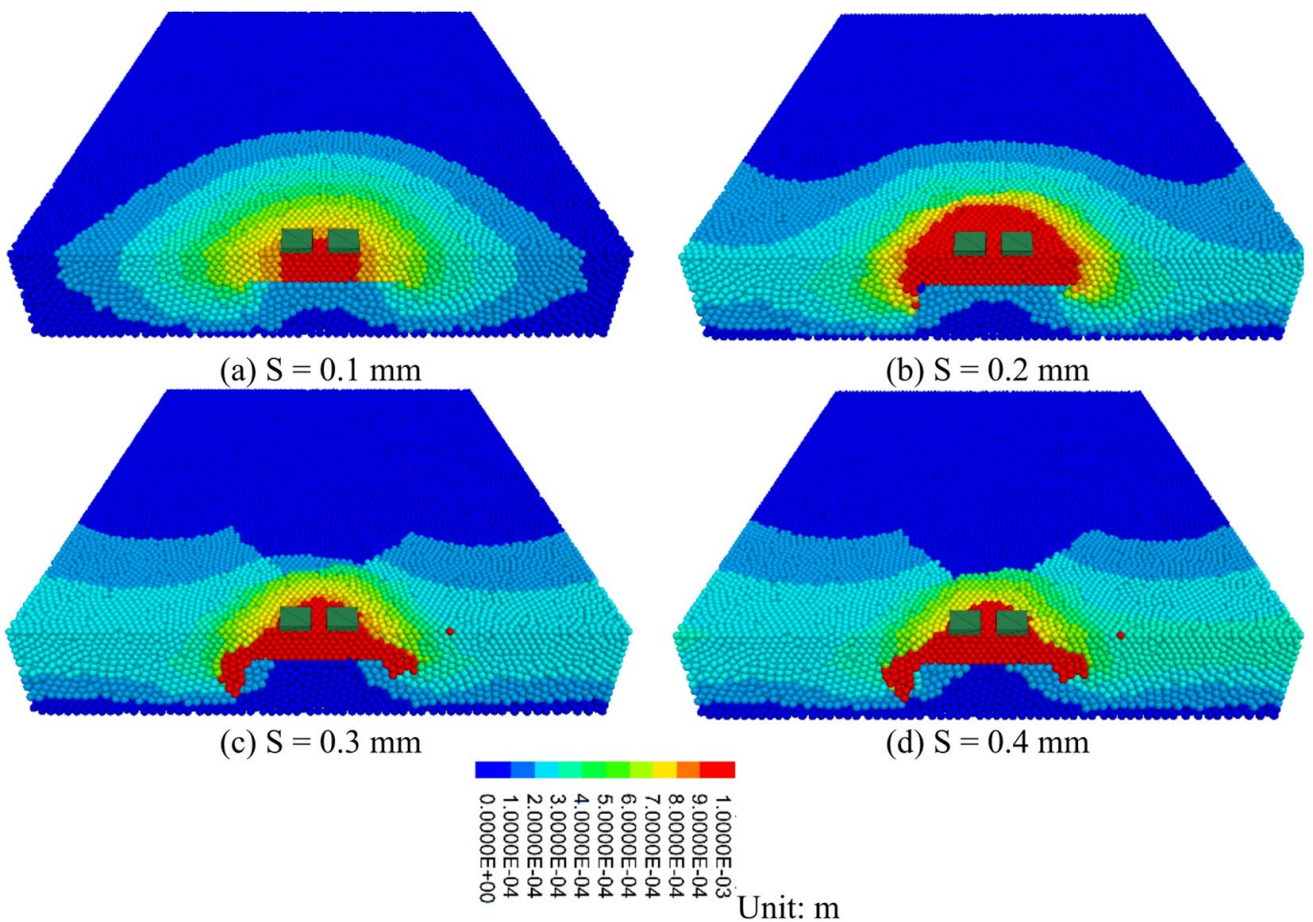
Displacement contour map of the road surface bottom plate void.

The Displacement contour map of the road surface bottom plate void are shown in Fig. 12. It can be observed that in the initial loading phase, the larger displacement area is beneath the loading plate, forming an elliptical pattern. This occurs because under the applied load, the concrete beneath the loading plate is first compressed, leading to a larger displacement in that area. With the increase in load, the displacement area continues to expand and stretches toward both sides of the void region. This occurs because the load is transferred through the concrete particles, generating stress concentration at the edges of the void, which leads to the displacement extending gradually toward both sides. Ultimately, failure occurs on both sides of the void region, forming a shear band. This failure pattern is caused by the concrete on both sides of the void region being subjected to significant shear stress under the load. When the shear stress exceeds the concrete’s shear strength, shear failure is formed. When the displacement reaches 0.2 mm, the range of particles with larger displacement is at its peak. This happens because the concrete has not yet fractured, and the load can effectively be transferred between particles, leading to significant displacement of a large number of particles. With continued loading, damage starts to occur between particles, interrupting the load transfer path. Once the bonding force between particles is broken, the load cannot be effectively transferred between the particles, reducing the displacement range. This phenomenon demonstrates the process by which concrete transitions from elastic deformation to plastic deformation and ultimately to failure under the applied load.

#### Crack propagation laws

**Fig. 13:**
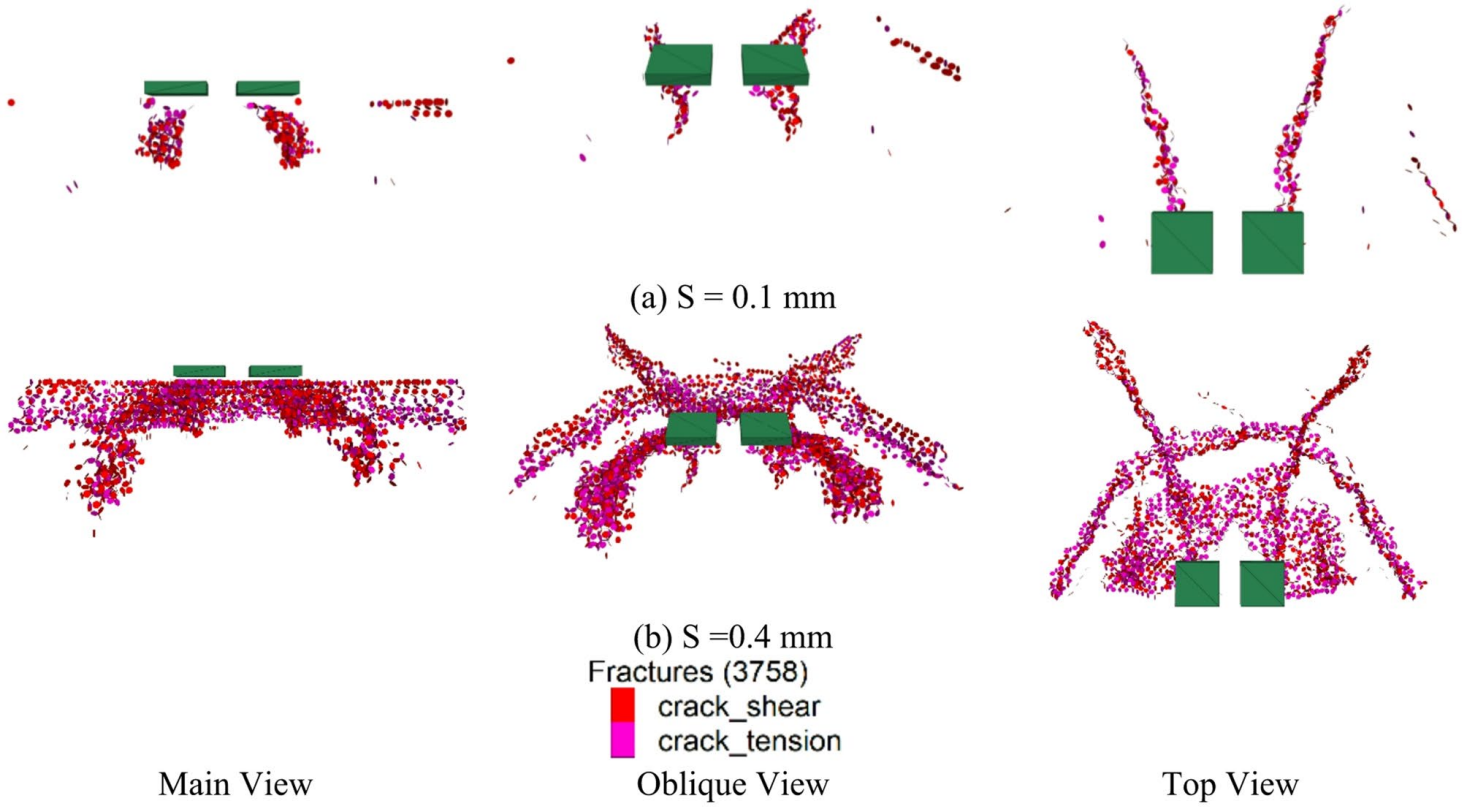
Distribution of cavity cracks on the road surface bottom plate.

The Fig. 13 shows that the cracks are divided into three distinct areas: Region 1 is located beneath the loading plate, primarily exhibiting compression fractures. Under the applied load, the concrete beneath the loading plate experiences considerable pressure, and when the pressure exceeds the concrete’s compressive strength, compression cracking occurs. The cracks in this area are mainly caused by vertical pressure. Region 2 consists of cracks that extend outward along the loading plate. These cracks are induced by tensile stresses as the load is transferred from the loading plate to the surrounding area. As the load spreads outward, cracks form in the concrete under tensile stress and propagate along the direction of load transfer. Region 3 features cracks along the shear plane, extending downward in a semi-circular distribution. This occurs because shear stresses are generated within the concrete under load, and when these stresses exceed the concrete’s shear strength, cracks develop along the shear plane. These cracks generally appear near the edges of the loading plate and the void area, creating a semi-circular distribution of cracks. Cracks primarily appear beneath the loading plate and extend outward from the loading plate region. This suggests that the generation and extension of cracks are closely related to the distribution and transmission of the load. The concrete beneath the loading plate is initially subjected to the load, causing compression cracks. As the load is transmitted outward, the cracks expand along the load transfer path. Crack propagation is predominantly observed in the area under the load, at the edges of the semi-circular region, and in the region where the load extends outward, exhibiting a high correlation with the displacement distribution. This is because, under loading, the displacement of concrete and the formation of cracks are closely interconnected.

#### Variation laws of the displacement of the ground foundation layer

**Fig. 14:**
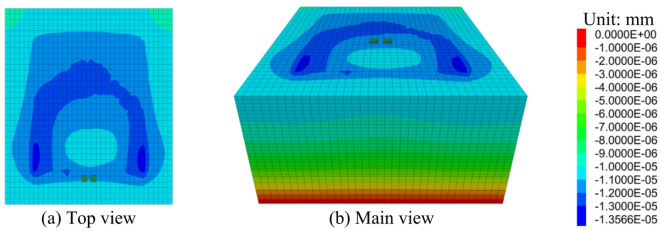
Displacement contour diagram of the base layer with void under the slab (S = 4 mm).

The displacement cloud map of the subbase of the cavitated road surface bottom plate (S = 4 mm) is shown in Fig. 14. From the figure, it can be observed that the displacement beneath the loading plate is smaller compared to the displacements on both sides of the void area. The primary cause of this phenomenon is the existence of the void area, which prevents the load from being directly transferred downward. Specifically, when the load is applied to the loading plate, the part of the plate above the void area, lacking support underneath, causes the load to diffuse toward the edges and surrounding areas of the void region. As a result, the concrete panels on both sides of the void area experience greater stress, leading to larger displacements. This pattern of displacement distribution is similar to that of crack distribution. In concrete panel structures, when voids appear, cracks typically form at the edges and around the void area. This happens because the stress concentration at the edges of the void area is more pronounced. When the load bypasses the void area and is transferred to the surrounding areas, the stress experienced by the concrete on both sides of the void increases gradually. Once the stress surpasses the tensile strength of the concrete, cracks will develop. As the load continues to increase, the cracks gradually expand. The direction and distribution of crack propagation are similar to that of displacement, both extending outward from the edges of the void area.

#### Distribution laws of the contact force chains

**Fig. 15:**
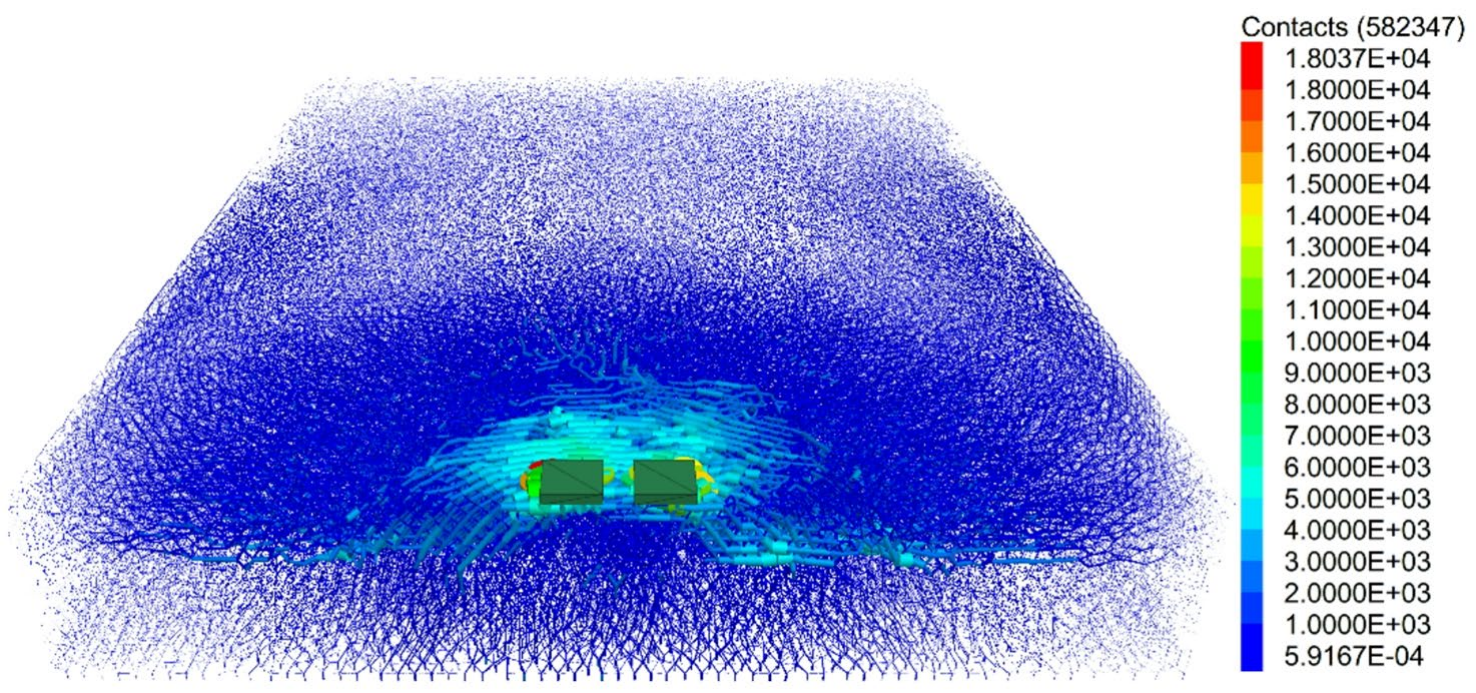
Distribution of emptying force chain of pavement baseplate.

Force chains refer to the channels through which forces are transmitted between particles, whereas strong force chains are the main transmission pathways for forces. Figure 15 shows the distribution of the force chains in the cavitated road surface bottom plate. From the figure, it can be seen that in the case of void beneath the slab loading, strong force chains are primarily distributed beneath the load and extend outward. Because of the existence of the void area, the distribution of force chains in the lower part of the void region is minimal. This is because there are no particles within the void area to transmit force, preventing the load from being directly transferred downward. As a result, the force chains bypass the void area to transmit forces. The distribution pattern of force chains aligns with the crack distribution pattern. Around the void region, force chains bypass the void area for transmission, resulting in stress concentration at the edges of the void region. This stress concentration phenomenon will cause cracks to form and propagate at the edges of the void region. Specifically, when the load is applied to the structure, the void area cannot bear the load, and the load is transferred through the surrounding particles, forming force chains. These force chains concentrate at the edges of the void region, causing large stresses. When the stress exceeds the material’s strength limit, cracks will form.

### Research on the loading mechanism of floor voiding after reinforcement

#### Establishment and calculation of reinforcement model

**Fig. 16:**
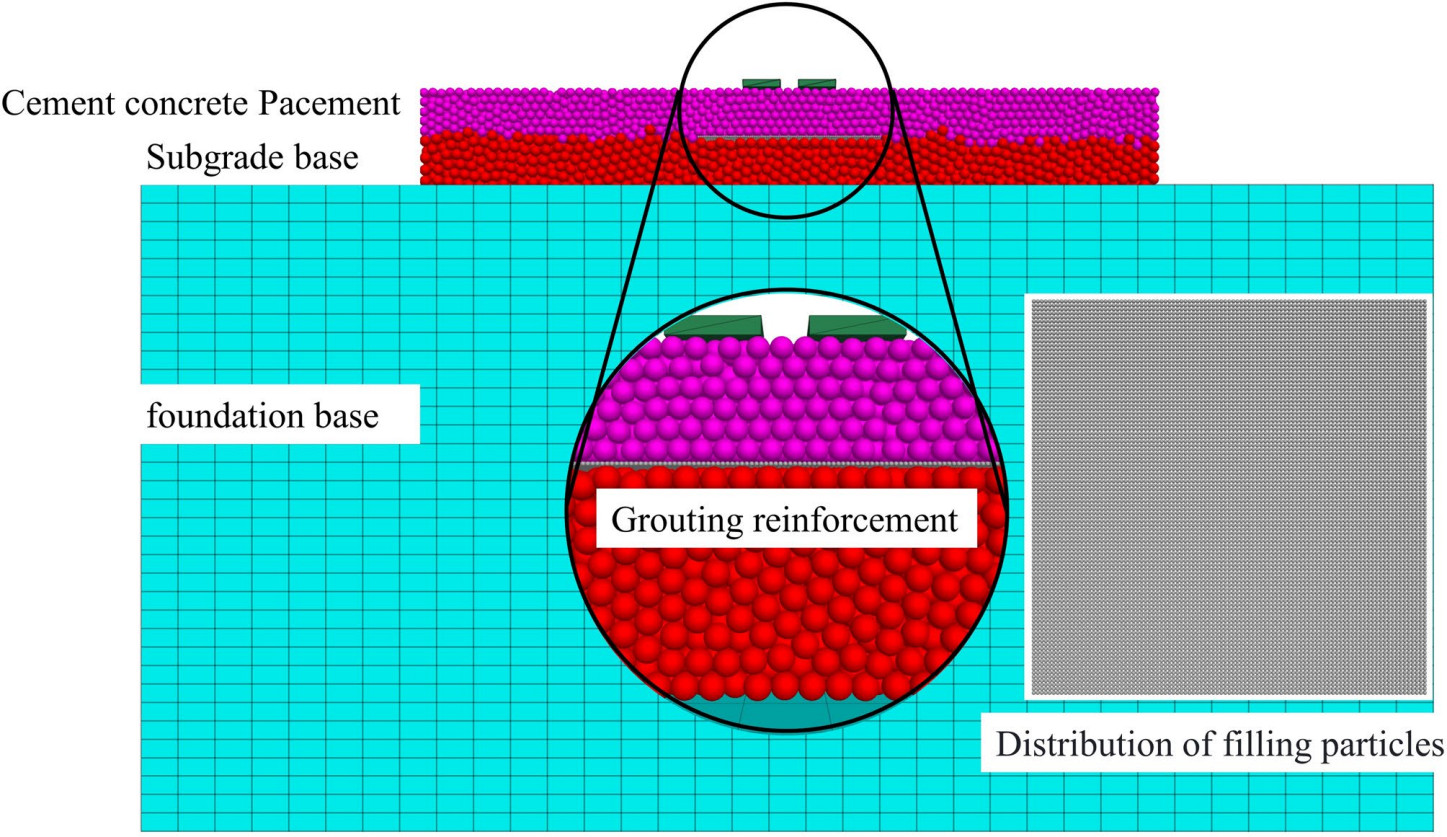
DEM of the bottom plate cavitation after reinforcement.

A reinforcement approach that uses generated particles for filling was employed in the void regions under the slab. This method uses small-radius particles injected into the void regions to achieve grouting reinforcement. Figure 16 shows DEM of the bottom plate cavitation after reinforcement. The particles in the reinforcement area have a diameter of 0.5 mm, and their total number is 9800. The smaller particle size in the reinforcement region allows for better filling of the voids within the empty space, enhancing the filling effectiveness. The fundamental principle of grouting reinforcement is to use injected particles to fill the void areas, creating a new support structure that enhances the load-bearing capacity and stability of the structure. The injected particles interact in the void region, forming a reinforced area with a specific strength. The mesoscopic parameters of the grouting material were chosen to be consistent with those of the original model, ensuring material compatibility in the simulation. To capture the interaction between the grouting material and the existing pavement structure, a parallel bond model was adopted, which effectively simulates the bonding effect at the interface, reflecting the collaborative mechanical behavior between the two.

#### Variation laws of load and microcracks after reinforcement

**Fig. 17:**
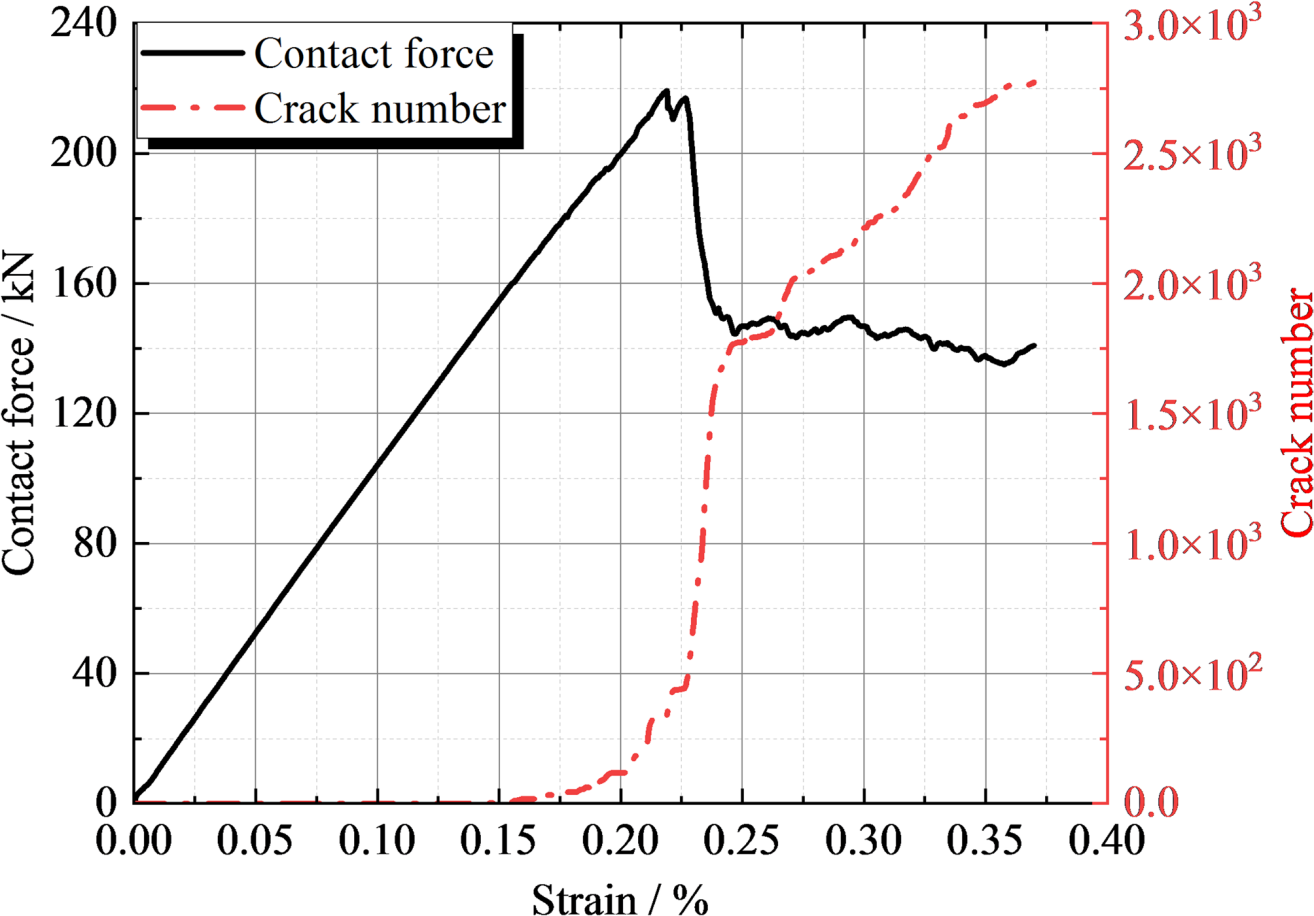
Curves of Load and Micro-Cracks Variation with Displacement after Reinforcement.

The curve of load and micro-cracks with displacement after reinforcement is shown in Fig. 17. From the figure, it can be observed that the peak occurs at a displacement of 0.225 with a peak strength of 220 kN. The curve shows overall brittle failure. The strength increased by 58.3% compared to the slab void. The displacement is in the elastic phase until 0.15 mm, which means that the deformation of the concrete pavement is elastic during this phase. When the load is removed, the structure can restore to its original shape. Micro-cracks start to appear in the structure when the load reaches 160 kN. At the peak, the crack count is about 500, while the peak crack number during slab void is 471, reflecting the number of internal micro-cracks under peak load. After the displacement reaches 0.25 mm, the rate of crack growth decreases. This could be due to the brittle failure of the concrete structure, leading to a rapid decrease in load, which results in a reduced crack growth rate.

#### Variation laws of the particle displacement field after reinforcement

**Fig. 18:**
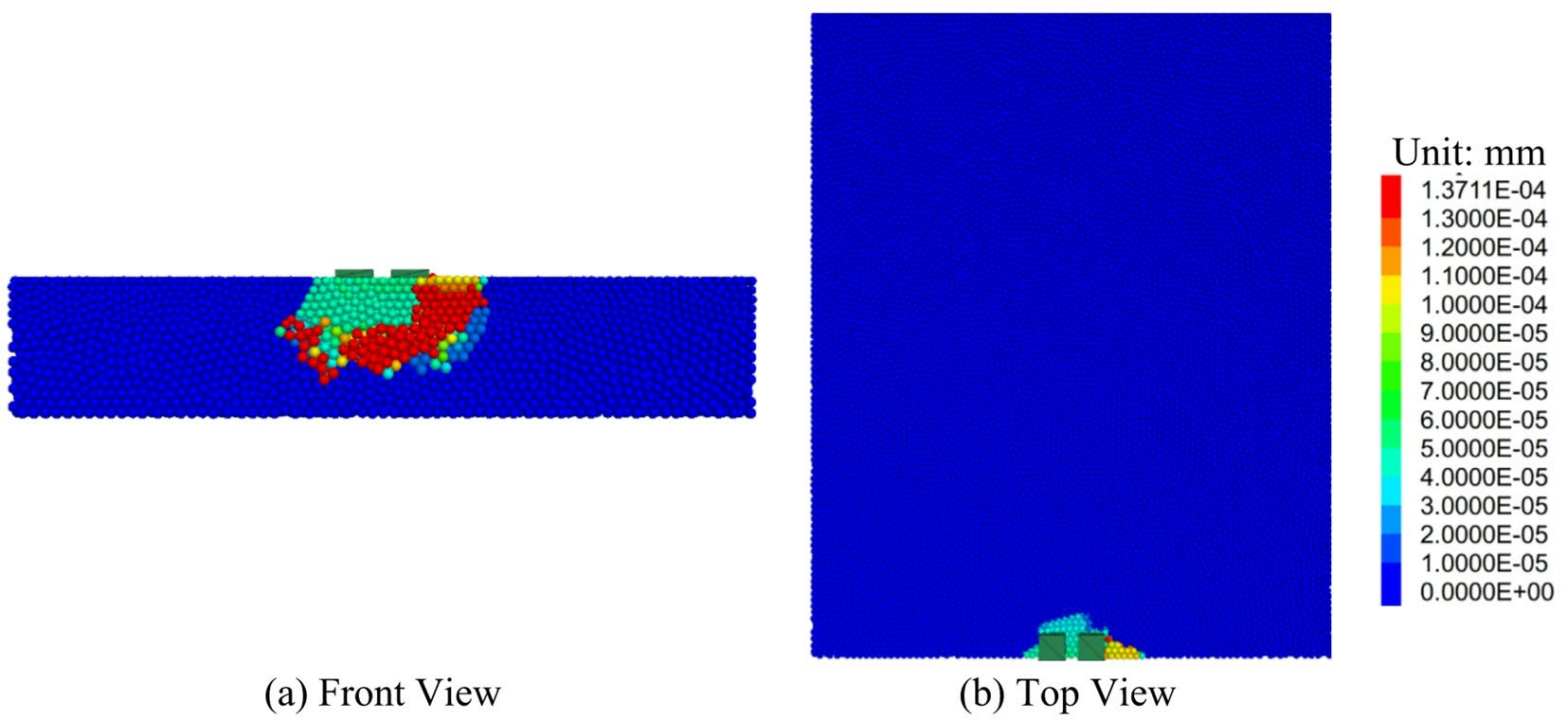
Cloud Map of Particle Displacement after Reinforcement(S = 0.4 mm).

The cloud map of particle displacement after reinforcement is shown in Fig. 18. From the figure, it can be observed that under the reinforced slab loading condition, the load in the large displacement area is transferred downward. This suggests that in this area, the load is effectively transmitted downward through the reinforced structure. The overall shear failure mode exhibits a triangular distribution. This triangular shear failure mode occurs due to the uneven stress distribution within the structure under loading. When the stress surpasses the material’s shear strength, a shear band forms in a particular region, resulting in structural failure. In the triangular distribution of shear failure, the shear band typically starts at the load application point and expands downward and to both sides. This is because during the load transfer process, stress concentration occurs within the structure, with the triangular area being the location of the most significant stress concentration.

#### Crack propagation laws after reinforcement

**Fig. 19:**
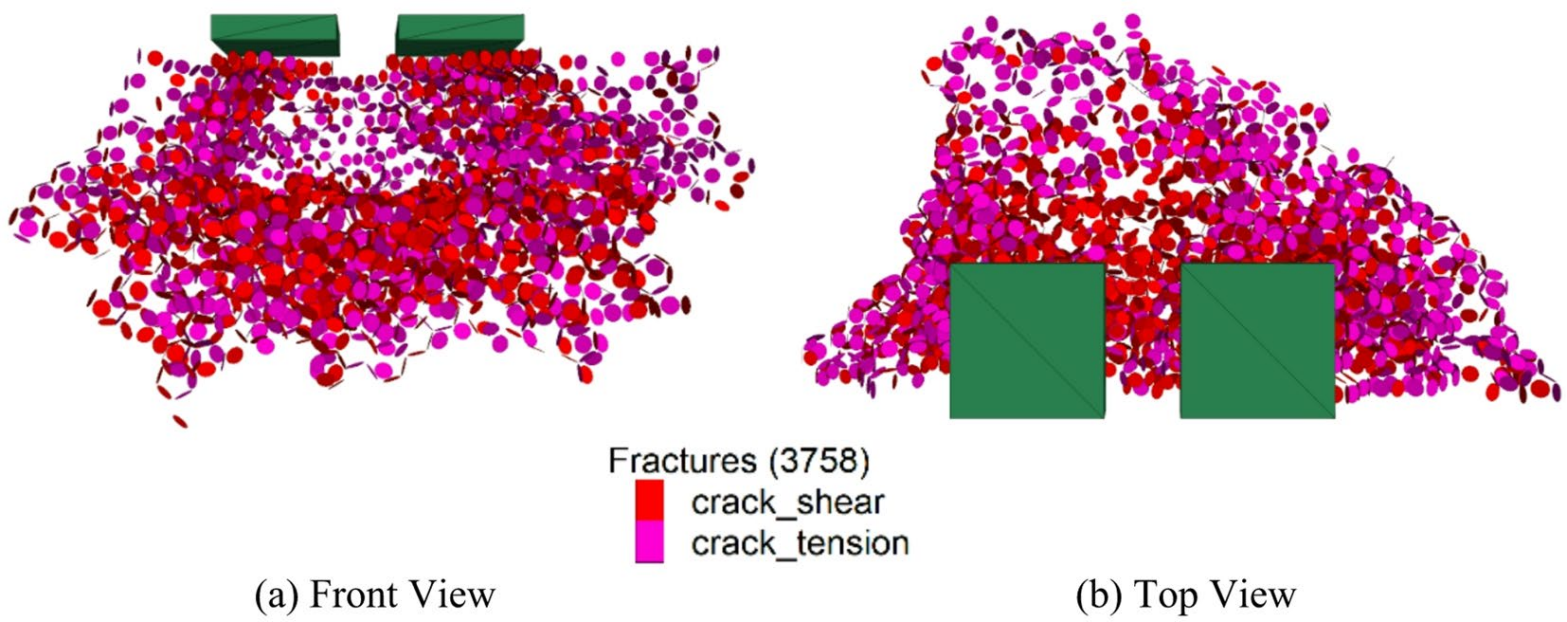
The Distribution Diagram of Cracks after Reinforcement.

From the post-reinforcement crack distribution map (Fig. 19), it can be seen that the cracks show a downward propagation trend, with greater crack depth. This phenomenon suggests that after the reinforcement of the bottom slab, the load can be transferred to the lower structure, causing the cracks to extend downward from the upper part. The cracks are generally distributed in a triangular shape, which is typically associated with stress concentration and the load transfer path. At the vertex of the triangle, stress concentration is more significant, causing the cracks to start from the apex and extend downward, forming a triangular crack pattern. Near the load application point, stress concentration is more intense, causing the cracks to form initially in this area. With the continuous application of the load, the cracks extend downward along the stress transfer path, resulting in deeper cracks. The triangular crack pattern reflects the manner in which the load is transmitted within the structure.

#### Variation laws of the displacement of the ground foundation layer after reinforcement

**Fig. 20:**
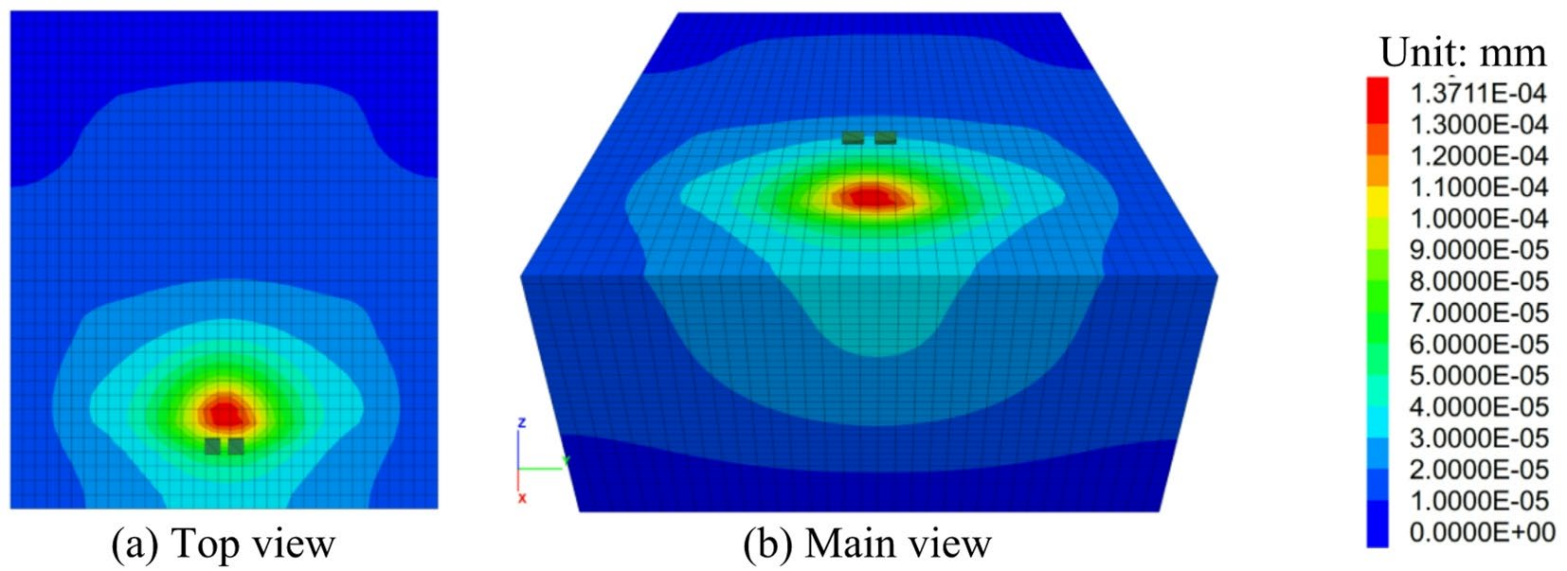
Displacement Contour Map of the Ground Foundation Layer of the Voided Floor Slab after Reinforcement (S = 4 mm).

From the post-reinforcement foundation displacement contour map (Fig. 20), it can be seen that the displacement primarily occurs near the loading plate. This is because during the loading process, the load is initially applied to the loading plate, causing displacement in the foundation near the loading plate. The maximum displacement is smaller than that under central loading conditions. This suggests that under side friction loading conditions, the load distribution and transmission in the foundation differ from those under central loading, resulting in a smaller displacement.

#### Distribution laws of the contact force chains after reinforcement

**Fig. 21:**
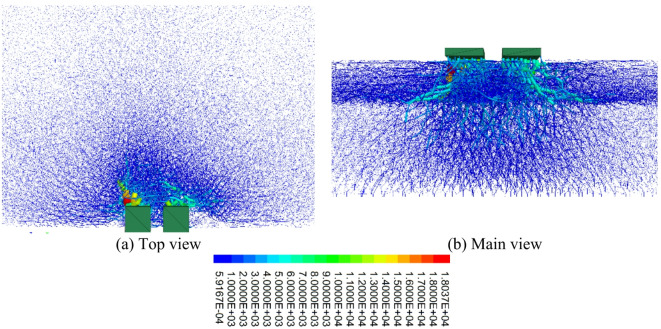
Distribution Diagram of Force Chains after Reinforcement (S = 0.4 mm).

From the post-reinforcement central loading force chain distribution diagram (Fig. 21), it can be observed that the force chains exhibit a downward transmission trend. In the concrete surface layer, the distribution of strong force chains is more concentrated. This suggests that in the reinforced structure, the load is transferred through the concrete surface layer. Because of its higher strength and stiffness, the concrete surface layer can efficiently transfer the load to the underlying structure. Compared to the base plate void condition, the force chain transmission range is broader after reinforcement. This is due to the reinforcement measures, which strengthen the overall integrity and bearing capacity of the structure, enabling the load to be more evenly distributed and transmitted. In the reinforced structure, the concrete surface layer plays a crucial load-bearing role. When the load is applied to the structure, it is initially borne by the concrete surface layer, then transmitted to the lower structure through the inter-particle force chains within the concrete. The concentrated distribution of strong force chains in the concrete surface layer indicates that this area is subjected to significant stress.

## Discussion

As the focus of this study is to compare the strength changes of concrete pavements before and after grouting, the grout material was considered as instantaneous in the model. This simplification is made to highlight the key research objective of evaluating the strength difference between the ungrouted and fully hardened grouted states, without involving the dynamic process of grout setting and hardening over time. Expansion of the current work in several directions is indeed intended: Firstly, vehicle cyclic loads will be adopted to simulate the actual stress state of the pavement, which can better reflect the influence of repeated loading on structural performance. Secondly, the time effect of grouting will be considered, and the strength of concrete panels with grouting materials of different curing times will be analyzed to explore the long-term performance of grouting reinforcement. Thirdly, the stress state of base slab voids under temperature loads will be investigated to assess the combined effects of environmental factors and structural defects.

## Conclusions

Based on the FEM-DEM coupling method, this study systematically investigates the void formation mechanism in concrete pavement base slabs and the strengthening effect of grouting reinforcement, providing critical insights into pavement structural behavior. The key findings and contributions are summarized as follows:

1. Model Validation and Construction: Mesoscopic parameters of concrete surface and base layers were accurately calibrated through uniaxial compression tests, explicitly demonstrating that macroscopic mechanical properties (e.g., compressive strength, elastic modulus) are significantly regulated by mesoscopic particle parameters (stiffness, friction coefficient) and contact constitutive relationships. Leveraging these calibrated parameters, an FEM-DEM coupling model was successfully constructed, with distinct discretization strategies: the base layer using particles of 3 cm radius, the concrete panel using particles of 2.5 cm radius, and the foundation layer employing finite element grids to balance computational efficiency and accuracy. The model’s simulated horizontal distribution of settlement displacements and the bottom-to-top dense-to-sparse transition of force chains were validated against field-measured soil stress data, confirming its validity and reliability. This model establishes a robust numerical platform for studying pavement structural interactions under complex loading conditions.
2. Base Slab Detachment Mechanism: Analysis of the load-displacement curve during base slab detachment reveals a clear three-stage failure process: rapid contact force increase to a peak of 139 kN, followed by a sharp decline, with crack initiation at ~ 76.6 kN (55% of peak load) and stabilization at ~ 3580 cracks at 0.26 strain. This progression directly maps the post-ultimate-bearing-capacity degradation of concrete structures. Loading displacement contours further illustrate that shear bands form bilaterally around the detachment area, with crack distribution tightly coupled to load transfer paths—specifically, smaller displacements under the load plate compared to the detachment area’s edges. The coordinated evolution of force chains and cracks, alongside shear band development, comprehensively unravels the multi-scale mechanical behavior driving base slab detachment, filling a gap in understanding how local voids propagate to structural failure.
3. Grouting Reinforcement Effect: Grouting reinforcement, achieved by filling the detachment area with 9800 particles of 0.5 mm diameter, markedly enhanced structural performance: peak strength reached 220 kN, representing a 58.3% increase in load-bearing capacity compared to the pre-reinforcement state, while retaining brittle failure characteristics consistent with concrete materials. The reinforced structure maintained elastic behavior up to 0.15 mm displacement, with crack initiation delayed to 160 kN and only ~ 500 cracks at peak load—far fewer than the unreinforced case—indicating suppressed damage evolution. Post-reinforcement, load transfer was effectively redirected downward, with shear failure exhibiting a triangular distribution, and crack depth increased but propagated in a more controlled manner. These results confirm that grouting not only boosts load-bearing capacity but also optimizes force distribution, offering a viable solution for mitigating pavement structural degradation.

The results indicate that in engineering practice, timely detection and treatment of base slab voids are crucial. If left unaddressed, the concrete panel is prone to damage under vehicle loads due to the existence of voids, as evidenced by the more rapid and extensive crack propagation in the ungrouted state. Additionally, the findings suggest that higher strength of the grouted concrete leads to better repair effects, as reflected by the slower crack development and reduced crack density in cases with higher grout strength.

## Data Availability

All data generated or analysed during this study are included in this published article. The datasets used and analysed during the current study available from the corresponding author on reasonable request.
